# A systematic review and meta−analysis on the prepectoral and partial subpectoral immediate single−stage Implant-Based Breast Reconstruction Using ADM

**DOI:** 10.3389/fonc.2026.1742423

**Published:** 2026-02-27

**Authors:** Tingjian Zhang, Yuyao Liu, Liang Huang, Qiang Zhang, Ying Li, Lingyan Du, Xueyun Zhao

**Affiliations:** 1 Department of Thyroid and Breast Surgery, The People′s Hospital of Leshan, Leshan, Sichuan, China; 2 Department of Radiology, The People′s Hospital of Leshan, Leshan, Sichuan, China

**Keywords:** acellular dermal matrix, breast reconstruction, immediate single-stage, postoperative complications, prepectoral, subpectoral

## Abstract

**Introduction:**

Acellular dermal matrices (ADMs) have made immediate single-stage prepectoral breast reconstruction (PBR) feasible and have promoted the application and development of partial subpectoral breast reconstruction (SBR). The type of mesh is regarded as a key factor influencing the safety of breast reconstruction. The optimal choice between prepectoral and partial subpectoral approaches for ADM-assisted immediate single-stage prosthetic breast reconstruction remains controversial. This study aimed to compare the safety profiles of these two surgical techniques.

**Methods:**

A systematic literature search was conducted in PubMed, Embase, Web of Science, and the Cochrane Library. All included patients underwent immediate single-stage implant-based breast reconstruction using ADM. Patient characteristics and postoperative complications were collected and summarized. Data analysis was performed using Cochrane RevMan and IBM SPSS software.

**Results:**

The meta-analysis incorporated 1,557 reconstructed breasts from eight eligible observational studies. Overall, PBR significantly reduced the total complication rate following immediate single-stage breast reconstruction compared with SBR. Additionally, PBR was associated with significantly lower rates of postoperative hematoma and skin–nipple necrosis compared with SBR.

**Conclusion:**

The meta-analysis demonstrated that PBR was associated with lower safety risks for ADM-assisted immediate single-stage implant-based breast reconstruction.

## Introduction

Breast cancer is the most common malignant tumor among women and poses a serious threat to both their physical and mental health (1). However, with advances in disease understanding and treatment methods, therapeutic outcomes have significantly improved, and survival rates have increased steadily over recent years. Nevertheless, the loss of one or both breasts can cause substantial physical and psychological distress, often leading to feelings of inferiority, depression, and difficulty reintegrating into society (2, 3). In recent years, efforts in early diagnosis and neoadjuvant therapy have markedly increased the rate of breast-conserving surgeries, making them a standard treatment option; however, many patients remain unsuitable for this approach. Breast reconstruction restores breast appearance, enhances psychological well-being, and significantly improves patients’ quality of life, without adversely affecting tumor prognosis (4–6). Breast reconstruction is generally classified into two main types: autologous reconstruction and implant-based reconstruction. Owing to its shorter operative time, reduced trauma, faster recovery, and superior aesthetic outcomes, implant-based reconstruction has become the predominant choice (7–9). Based on the plane of implant placement, implant-based reconstruction is categorized into PBR and subpectoral SBR techniques. In PBR, the implant is positioned above the pectoralis major and serratus muscles following mastectomy to preserve the natural breast contour.

SBR involves placing the prosthesis between the pectoralis major muscle and the chest wall, requiring partial dissection of the pectoralis major muscle. Alternatively, the implant may be positioned partly beneath the pectoralis major muscle and partly beneath the lower mastectomy flap, creating a dual-plane configuration. In recent years, the introduction of ADM and synthetic meshes promoted the advancement of implant-based breast reconstruction techniques (10, 11). The SBR dual-plane technique is now widely applied in clinical practice because it reduces implant exposure, displacement, and rippling while achieving superior aesthetic outcomes compared with traditional SBR (12–14). However, detachment of the pectoralis major muscle may exacerbate postoperative pain and spasms, cause animation deformities, and impair upper limb function (15, 16). PBR offers several advantages, including reduced postoperative pain, minimal functional impairment, elimination of animation deformities, and more natural aesthetic outcomes (12, 17–19). Consequently, PBR has gained increasing popularity among both patients and surgeons. Nevertheless, the safety of PBR remains controversial because limited soft tissue coverage may lead to complications such as implant exposure, capsular contracture, and implant edge visibility (17, 20, 21). Furthermore, large randomized controlled trials and meta-analyses directly comparing the safety of prepectoral and partial subpectoral immediate single-stage breast reconstruction are still lacking. In addition, because the prepectoral technique has only recently been reintroduced, previous systematic reviews included limited literature and had relatively short follow-up periods. Therefore, these reviews may not accurately assess the true incidence of delayed complications following PBR. Additionally, the inclusion criteria for some systematic reviews did not strictly limit the timing or approach of surgery (e.g., delayed reconstruction, two-stage techniques, mixed techniques, etc.), which may influence the analysis of surgical safety. Immediate single-stage prepectoral and partial SBR procedures often require mesh reinforcement. However, numerous studies have shown that the safety profile of ADM differs from that of other meshes, and mesh selection is a critical variable influencing reconstruction safety.

Moreover, no large randomized controlled trials have yet determined whether safety differs among various mesh types used in breast reconstruction (22–24). Previous meta-analyses frequently overlooked the influence of mesh type, a key factor affecting study outcomes. For example, some included studies used ADM, others employed TiLOOP Bra or different meshes, while some used none at all, leading to reduced reliability and confidence in the pooled results. To address this issue, we conducted a study to compare the safety of prepectoral and partial subpectoral immediate single-stage implant-based breast reconstruction using ADM.

## Methods

### Searching strategy

Comprehensive search of PubMed, EMBASE, Web of Science and Cochrane Library for relevant studies published from relevant studies published to June 21, 2025 according to PRISMA guidelines. We used a combination of controlled vocabulary terms (e.g., MeSH) and free-text keywords, and no filters or limits were applied during the searches. The search terms were:(((((acellular dermis[MeSH Terms]) OR (acellular dermal matrix)) OR (ADM)) AND ((mammaplasty[MeSH Terms]) OR (breast reconstruction))) AND ((prepectoral OR suprapectoral OR subcutaneous OR premuscular OR supramuscular OR muscle-sparing OR pectoralis-sparing) AND (subpectoral OR submuscular OR retropectoral))) AND ((((prostheses and implants[MeSH Terms]) OR (implant)) OR (prosthesis)) OR (prosthetic)). In addition, references to relevant studies were reviewed for potential inclusion. The selection of studies was based on the following criteria: Inclusion criteria: (1) Direct implantation of prepectoral/partial subpectoral breast reconstruction after mastectomy;(2) Studies describing the use of ADM in prosthetic breast reconstruction and specifying that it is used in both prepectoral and partial subpectoral breast reconstruction;(3) Studies reporting the incidence of postoperative complications;(4) Randomized controlled trials or prospective/retrospective cohort studies. Exclusion criteria: (1) No direct implantation of prosthesis after mastectomy (implant expander was used in the operation, the timing of surgery was delayed reconstruction, the surgical approach was autologous reconstruction.);(2) Study did not provide a detailed description of ADM in both groups of patients;(3) Literature review without original data, case reports, reviews, conference abstracts, animal experiments, guidelines, expert discussions, consensus, visualization of surgical papers or patents, etc. (4) The total sample size for the study is no less than 20 reconstructed breasts, with each study cohort comprising no less than 10 reconstructed breasts.;(5) Articles that do not provide complete information. This study was not prospectively registered in PROSPERO.

### Definition of outcome indicators

Seroma: A sterile fluid collection within the periprosthetic pocket or surgical field. The fluid typically consists of lymphatic fluid, inflammatory exudate, and interstitial fluid. Clinically, it may present as breast swelling or a localized fluctuant bulge, occasionally with mild pain or tenderness, and generally without systemic signs of infection such as fever, erythema, or warmth. Wound dehiscence: Partial or complete separation of the surgical incision due to factors such as local ischemia, excessive wound tension, or inadequate soft-tissue coverage, resulting in failure of normal wound healing and reopening of the incision. Capsular contracture: Pathologic thickening and contraction of the fibrous capsule surrounding the implant, which may lead to breast firmness, distortion, and pain, thereby compromising both implant function and aesthetic outcome. Severity is commonly graded using the Baker classification. Infection: Postoperative infection of the surgical site caused by bacterial or other pathogenic organisms, typically characterized by localized erythema, swelling, pain, and increased temperature, with possible drainage or purulence. Severe cases may involve the implant or periprosthetic space. Rippling: Visible or palpable surface irregularities of the reconstructed breast, often described as a “step-off” or undulating contour, most commonly observed at the transition between the implant edge and the chest wall, particularly in the upper pole. Animation deformity: Implant displacement and breast contour distortion induced by pectoralis muscle contraction, typically manifesting as abnormal movement of the reconstructed breast during upper extremity activity; displacement commonly occurs in a superolateral direction depending on implant plane and muscle dynamics. Skin–nipple necrosis: Compromised perfusion of the mastectomy skin flap and/or nipple–areolar complex following surgery. Inadequate blood supply or excessive tension may result in tissue necrosis, clinically presenting as progressive discoloration (dusky appearance to black eschar) and decreased local skin temperature. Implant loss: Removal of the breast implant due to complications (e.g., infection, exposure, severe necrosis) or inability to maintain the implant in an appropriate anatomic position, resulting in failure of implant retention. Hematoma: A collection of blood within the surgical pocket or surrounding tissues, typically presenting with acute swelling, pain, and ecchymosis; drain output may appear bright red, and significant hematoma may necessitate evacuation.

### Data extraction

Data extraction was piloted by one author. Then, data were independently extracted and entered into a standard Microsoft Excel data collection template by two authors. Any disputes will be adjudicated with the involvement of the third author, and ultimately resolved through joint discussion among all three authors. We collected relevant information, including but not limited to: study characteristics, first author, year of publication, country of authorship, study design, study duration, number of patients, number of reconstructed breasts, age, BMI, preoperative information, and postoperative complications. The quality of the study was evaluated using the Newcastle-Ottawa Scale (NOS). When disagreements arose, the three authors discussed and resolved them together.

### Data analysis

Data were pooled and analyzed using Microsoft Excel and Cochrane RevMan Version 5.4 (Cochrane Collaboration, Copenhagen). Complication rates were compared between prepectoral and subpectoral reconstruction. Odds ratios (ORs) with 95% confidence intervals (CI) were calculated using the Mantel-Haenszel test. The number of reconstructed breasts was used in the meta-analysis for each complication. For studies that only reported results per patient rather than per reconstructed breast, we assumed that the number of reconstructed breasts was equal to the number of patients, as each patient had at least one breast reconstructed. In addition, since the total number of breasts was difficult to determine without laterality information, and the overwhelming majority of reconstructions in our dataset were unilateral, assuming bilateral reconstruction was inappropriate. Potential heterogeneity across studies was assessed using the Cochrane Q statistic and the *I*^2^ test, with p<0.1 or *I*^2^>50% indicating heterogeneity. Fixed-effects models were used for homogeneous datasets and random-effects models for heterogeneous datasets. Sensitivity analysis was performed for all outcomes. If the overall-complication outcome is clinically comparable across studies and statistical heterogeneity is low, the primary meta-analysis will pool effect estimates using a fixed-effect model. In parallel, a random-effects model will be applied as a sensitivity analysis, and under the random-effects framework, we will calculate the 95% prediction interval (PI) to reflect the plausible range of the true effect that may be observed in future studies. For outcomes informed by two or fewer studies, pooled estimates may be insufficiently robust and should be interpreted with caution. We therefore conducted a random-effects sensitivity analysis in addition to the fixed-effect model, and we present these outcomes narratively as exploratory findings only, rather than as definitive results.

## Results

### Study characteristics

**Figure 1:**
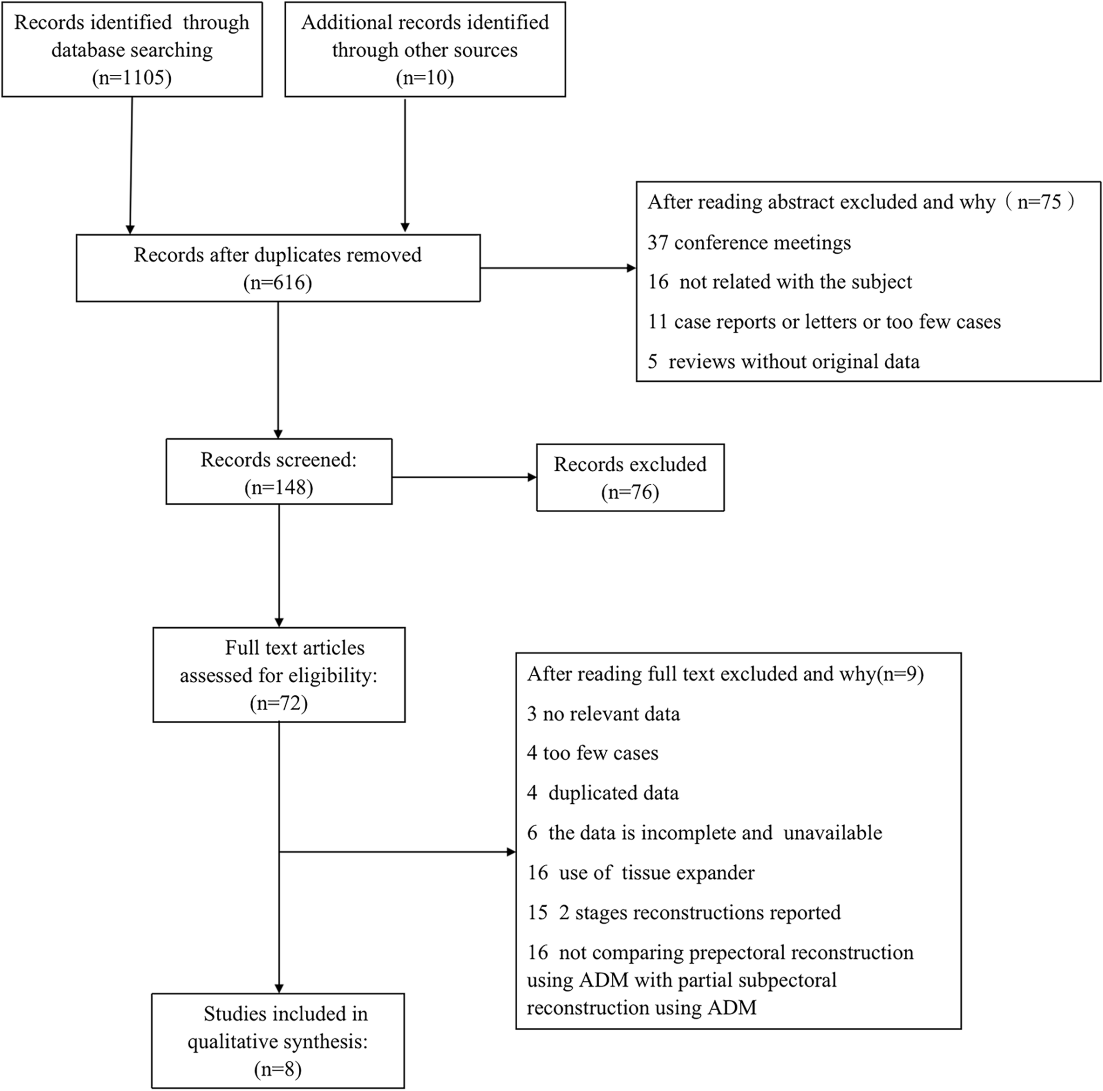
Flowchart demonstrating selection process for included studies. Meta-analysis data were collected following the PRISMA (preferred reporting items for systematic reviews and meta-analyses) guidelines.

A total of 1115 articles were retrieved from various databases, and 72 full-text articles were screened by excluding duplicates and after reading the abstracts. We evaluated the 72 full-text articles according to the inclusion and exclusion criteria. A total of 8 studies were finally considered adequately eligible (**Figure 1**). Four of these studies were prospective, and the others were retrospective (**Table 1**). These eight studies included a total of 1290 patients with a total of 1557 reconstructed breasts. All breasts were reconstructed with immediate postoperative implant placement, and all used acellular dermal matrix. Postoperative follow-up of all patients was more than 3 months. Postoperative complications of breast reconstruction are shown in **Table 2**. The overall research quality of the included articles was high, with scores of 6 and above. (**Table 3**).

**Table 1:** Study characteristics of the included studies.

Studies (author, year)	Study design	Reconstructed plane	No. of patients	Mean age ± SD.year	Mean BMI ± SD.kg/m^2^	Follow-up time ± SD.month
Benjamin G (2018)	Prospective cohort	Prepectoral	28	47.5 (36-55)	26 ± 4.0	>3
		Subpectoral	12	48 (31-51)	23.4 + 4.8	>3
John Mathew (2021)	Prospective cohort	Prepectoral	85	48 (27-73)	26 (19-46)*	24*
		Subpectoral	24	47 (28-63)	27 (19-48)*	44*
Joon Seok (2021)	Prospective cohort	Prepectoral	20	46.2 ± 7.1	20.93 ± 2.05	6
		Subpectoral	14	46.8 ± 4.4	21.28 ± 1.62	6
Shayda J (2019)	Prospective cohort	Prepectoral	62	54	27	>3
		Subpectoral	67	48	26	>3
Jeong-Hoon (2024)	Retrospective cohort	Prepectoral	53	47.68 ± 7.45	23.92 ± 3.61	>3
		Subpectoral	114	46.56 ± 9.65	22.65 ± 2.81	>3
Glenda Giorgia (2020)	Retrospective cohort	Prepectoral	39	39	22.65 ± 2.81	6
		Subpectoral	55	55		6
Diego Ribuffo (2020)	Retrospective cohort	Prepectoral	172	55.72 ± 4.5	25.36 ± 2.69	16.5
		Subpectoral	470	56.20 ± 7.6	24.60 ± 3.85	27.8
Oscar J (2019)	Retrospective cohort	Prepectoral	33	54 (45-62)	25.8	20.3 *
		Subpectoral	42	47 (40-60)	24.9	21*

*Median ± interquartile range.

**Table 2:** Summary of postoperative complications of the two cohort.

Incidences of complications	No. of studies	Prepectoral (% per breast)	Subpectoral (% per breast)
Seroma	8	37/629 (5.88%)	65/928 (7.00%)
Hematoma	6	9/566 (1.59%)	35/895 (3.91%)
Implant loss	7	17/575 (2.96%)	30/861 (3.48%)
Capsular contracture	4	27/335 (8.06%)	42/706 (5.95%)
Skin-nipple necrosis	6	19/337 (5.64%)	38/395 (9.62%)
Animation deformity	2	0/227 (0.00%)	352/523 (67.30%)
Wound dehiscence	2	5/262 (1.91%)	14/578 (2.42%)
Rippling	2	14/105 (13.33%)	1/38 (2.63%)
Infection	8	22/629 (3.50%)	38/928 (4.09%)
Total complications	8	150/629 (23.85%)	291/928 (31.36%)

**Table 3:** Newcastle–Ottawa grading scale.

Study	Score
Benjamin G (2018)	8
John Mathew (2021)	7
Joon Seok (2021)	6
Shayda J (2019)	7
Jeong-Hoon (2024)	6
Glenda Giorgia (2020)	8
Diego Ribuffo (2020)	7
Oscar J (2019)	7

### Synthesis of the results

Separate meta-analyses were performed on the incidence of implant loss, seroma, capsular contracture, hematoma, infection, skin-nipple necrosis, wound dehiscence, rippling and total complication rates. No heterogeneity was found in all analyses (all *p*>0.1 & *I*²<50%) (**Table 4**). Therefore, a fixed-effects model was used for all. Pooled analysis of the rate of capsular contracture in the reconstructed breasts in the four studies found no statistically significant difference in the rate of capsular contracture between the prepectoral and subpectoral groups. Similarly, seven studies have reported on the implant loss rate after prepectoral and partial subpectoral breast reconstruction, and pooled analyses found comparable rates of implant loss between the two groups. Only two studies reported on the incidence of rippling and wound dehiscence, and the results demonstrated no statistically significant difference in the incidence of rippling and wound dehiscence between the two groups. In addition, our study found no statistical difference in the rates of smoking, neoadjuvant chemotherapy, and postoperative radiotherapy between the two groups(**Table 5**). Due to the limited data available, we did not capture information on revision procedures or aesthetic refinements/adjustments.

**Table 4:** Summative forest plot for primary post-operative endpoints.

Complication	Odds ratio* (95% CI)	*P*	*I*^2^ (%)	*Ph*
Seroma	0.83 [0.54, 1.28]	0.40	0%	0.80
Hematoma	0.44 [0.21, 0.91]	0.03	8%	0.36
Implant loss	0.97 [0.51, 1.84]	0.93	0%	0.75
Capsular contracture	1.33 [0.80, 2.20]	0.27	0%	0.27
Skin-nipple necrosis	0.50 [0.28, 0.89]	0.02	0%	0.46
Infection	0.83 [0.48, 1.43]	0.50	44%	0.10
Wound dehiscence	0.81 [0.28, 2.28]	0.68	0%	0.74
Rippling	3.80[0.64, 22.48]	0.14	43%	0.19
Total complications	0.70 [0.55, 0.90]	0.004	27%	0.21

*P*-values for ORs; *Ph* values of the Q-test for heterogeneity test; *I*^2^ refers to the proportion of total variation due to between-study heterogeneity.

*Means the OR of the PBR group/SBR group.

**Table 5:** Supplementary analysis results under the random-effects model.

Complication	No. of studies	Relative risk* (95% CI)	PI (95% CI)	*P*	*I*^2^ (%)	*Ph*
Seroma	8	0.86 [0.58, 1.28]	[0.51, 1.45]	0.45	0%	0.80
Hematoma	6	0.46[0.20, 1.06]	[0.11, 1.95]	0.07	8%	0.37
Implant loss	7	0.99 [0.53, 1.87]	[0.41, 2.42]	0.99	0%	0.75
Capsular contracture	4	1.30 [0.82, 2.07]	[0.47, 3.59]	0.26	0%	0.74
Skin-nipple necrosis	6	0.54[0.32, 0.94]	[0.25, 1.16]	0.03	0%	0.53
Infection	8	0.84 [0.37, 1.91]	[0.10, 6.84]	0.67	43%	0.11
Wound dehiscence	2	0.81 [0.29, 2.27]	NR^#^	0.69	0%	0.74
Rippling	2	2.28[0.18, 28.71]	NR^#^	0.52	42%	0.19
Total complications	8	0.81 [0.65, 1.02]	[0.49, 1.35]	0.08	30%	0.19

*P*-values for RRs; *Ph* values of the Q-test for heterogeneity test; *I*^2^ refers to the proportion of total variation due to between-study heterogeneity *Means the RR of the PBR group/SBR group.

**^#^** NR: Not reported. The outcome measures of wound dehiscence and rippling were only included in two studies, so we did not calculate predicted interval values.

### Hematoma

**Figure 2:**
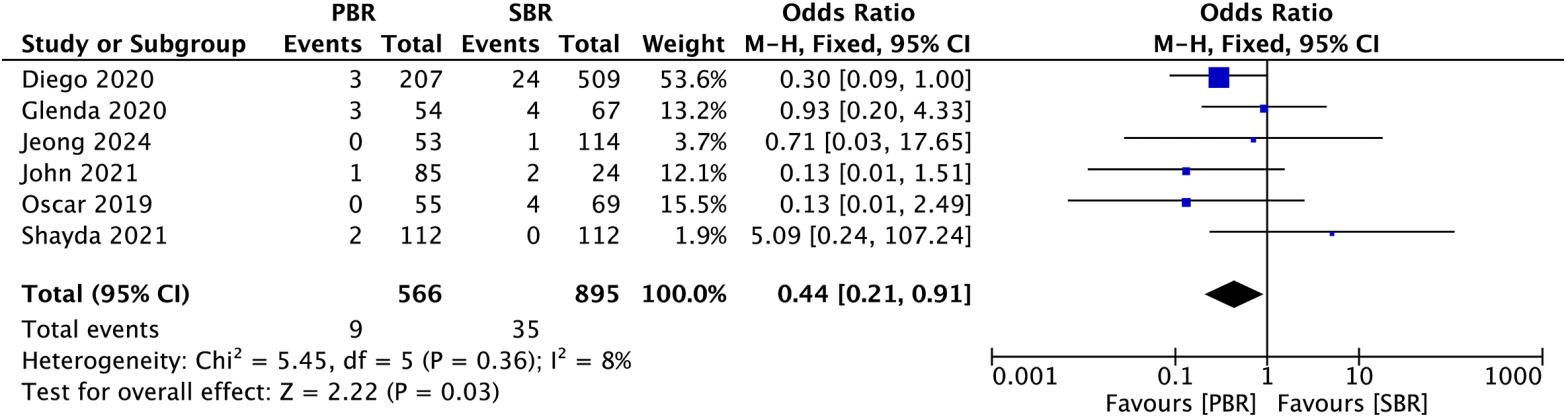
Efficient rates of hematoma prevention in patients receiving PBR or SBR (fixed effects model). The diamond represents the fixed effects odds ratio, and the width of the diamond corresponds to the 95% confidence interval.

A total of 6 studies (n: 1461 breasts, PBR: 566, SBR: 895) reported the effect of surgical method on the incidence of postoperative hematoma. The incidence of hematoma was 1.59% in the PBR group and 3.91% in the SBR group. The absolute risk differential(ARD)is 2.32%. Pooled analysis of these six studies demonstrated that the rate of postoperative hematoma was significantly lower in the prepectoral group than in the subpectoral group, representing a reduction of 2.32%.(OR: 0.44, 95% CI: 0.21–0.91,p = 0.03; **Figure 2**).

### Skin-nipple necrosis

**Figure 3:**
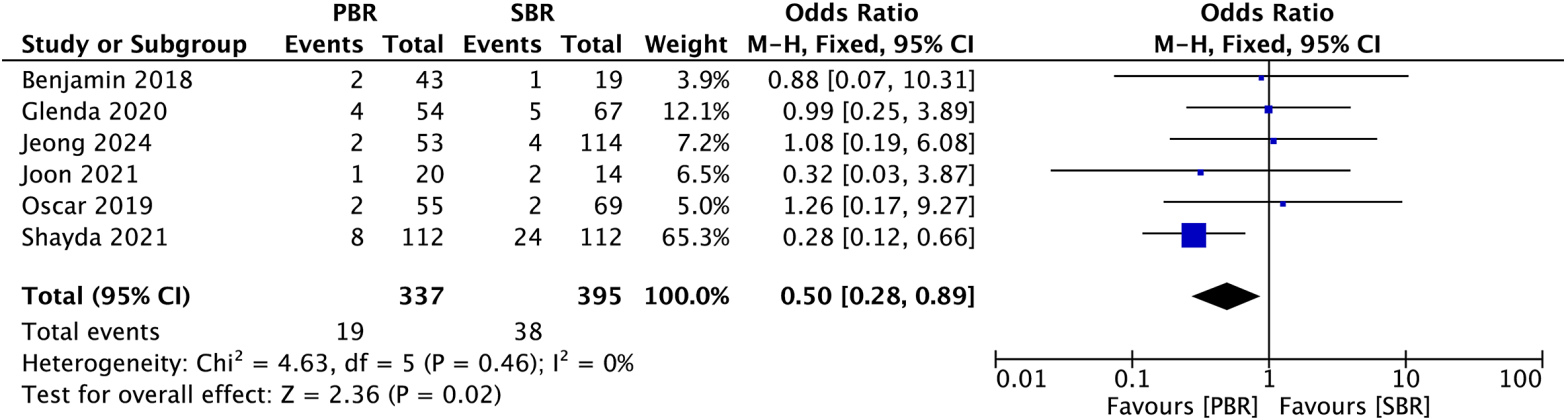
Efficient rates of skin-nipple necrosis prevention in patients receiving PBR or SBR (fixed effects model). The diamond represents the fixed effects odds ratio, and the width of the diamond corresponds to the 95% confidence interval.

A total of six studies (n: 732 breasts, PBR: 337, SBR: 395) reported the rate of skin-nipple necrosis in the PBR and SBR groups. The rate of skin-nipple necrosis was 5.64% in the PBR group and 9.62% in the SBR group. The ARD is 3.98%.Our pooled analysis of these six studies showed a significant reduction in postoperative skin- nipple necrosis in patients undergoing PBR compared with the SBR group, representing a reduction of 3.98%(OR: 0.50, 95% CI: 0.28-0.89, p=0.02; **Figure 3**).

### Total complications

**Figure 4:**
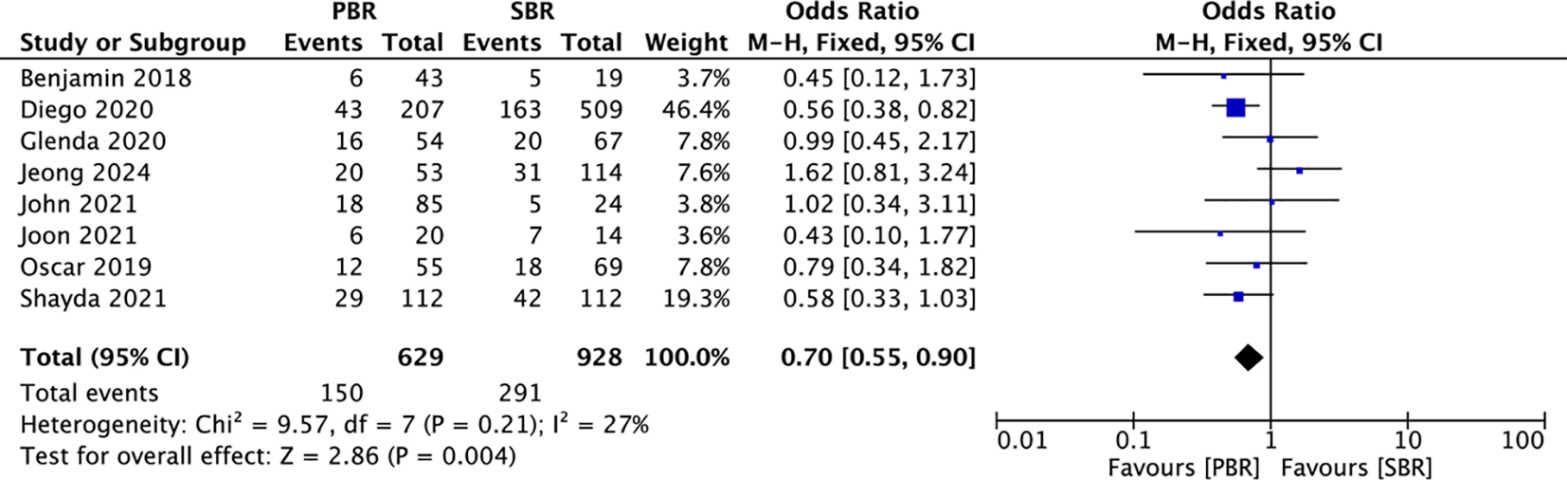
Efficient rates of total complication prevention in patients receiving PBR or SBR (fixed effects model). The diamond represents the fixed effects odds ratio, and the width of the diamond corresponds to the 95% confidence interval.

Statistical analyses of the total complication rates of patients in the PBR and SBR groups in these eight studies were performed. The total complication rate in the PBR group was 23.85% (150/629) compared with 31.36% (291/928) in the SBR group. The ARD is 7.51%. The meta-analysis showed a lower complication rate in patients receiving PBR compared to the SBR group, representing a reduction of 7.51%. (OR: 0.70, 95% CI: 0.55-0.90, *p* = 0.004; **Figure 4**).

### Animation deformity

Only two studies reported postoperative animation deformity, and both compared subpectoral (SBR) versus prepectoral (PBR) implant placement (25, 26).Across these studies, no events were observed in the PBR group (0/227), whereas animation deformity was frequent in the SBR group (352/523; 67.3%). Given the very small number of contributing studies and the presence of a zero-event arm, we did not perform a conventional pooled meta-analysis. Instead, we provided a narrative synthesis and a study-level effect estimate based on risk difference (RD). The absolute risk difference consistently favored PBR (RD ≈ −0.67 overall), indicating a markedly lower risk of animation deformity with PBR across the included evidence. Interpretation should be cautious because these two studies had differing follow-up periods for their results, and there were also some influential factors we could not exclude (such as implant type, technical details, and flap thickness), which could influence the observed event rates.

### Supplementary analysis results under the random-effects model

The fixed-effect primary analysis estimates a common effect, whereas the prediction interval (PI) reflects the range of effects that may be observed across different settings. Based on the 95% PI calculated under the random-effects model in our supplementary analyses, all PIs crossed the line of no effect, suggesting that the true effect in future studies may range from benefit to null; therefore, the reproducibility and generalizability of the findings may be limited (**Table 5**). For wound dehiscence and rippling, only two studies were included. Given the small number of studies, we did not report prediction intervals for these outcomes, and the pooled estimates are unstable and imprecise; thus, the results should be interpreted with caution.

### Publication bias

**Figure 5:**
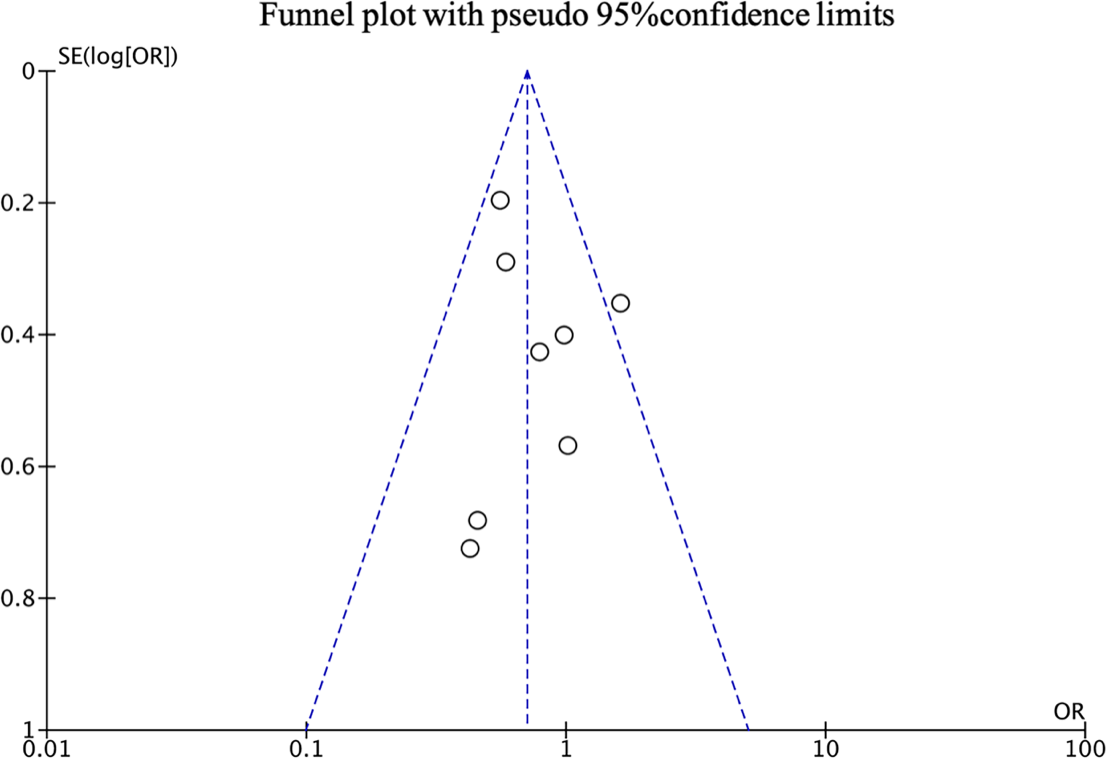
Funnel plot showed that the studies were symmetrically distributed on both sides of the vertical dashed line, concentrated at the top, and mostly located within the diagonal dashed line. Funnel plot demonstrated the absence of publication bias among the studies on the total complications. Vertical dashed lines represent the combined effect size, and diagonal dashed lines represent the 95% confidence limit. SE, standard error. OR, odds ratio.

The total complications funnel plot is shown in **Figure 5**. From visual inspection, most points are inside the funnel. **Supplementary Figure S1** provides funnel plots for each meta-analysis. Overall, they have good symmetry, indicating no evidence of publication bias.

### Sensitivity analysis

Sensitivity analyses were used in the current meta-analysis to assess the robustness and reliability of the combined results. In all of our analyses, omitting each study individually had no significant effect on the results. Therefore, the results of the sensitivity analysis showed that the meta-analysis was reliable and stable. (**Supplementary Table S1**).

## Discussion

As the survival rate of breast cancer continues to improve, both physicians and patients are increasingly emphasizing the quality of life after treatment. Consequently, the demand for breast reconstruction has risen, accompanied by significant advances in reconstructive techniques (27). For decades, surgeons have sought the optimal plane for prosthetic implantation to minimize complications and achieve more aesthetically pleasing outcomes. In the 1970s, Snyderman and Guthrie first attempted PBR. However, this early approach was associated with a high failure rate, primarily due to poor post-mastectomy flap quality and the absence of durable implant support materials. Consequently, a gradual transition occurred from PBR to SBR (28–30).With the advent of ADM and synthetic mesh, the technique of posterior pectoral muscle reconstruction has been improved. It also facilitated the application and development of the posterior pectoralis muscle dual plane technique, which significantly improved the inferior pole projection of the breast and improved the clarity and aesthetics of the inframammary fold (31, 32).

Nevertheless, many patients still experience complications caused by pectoralis major contraction, including suboptimal implant projection, animation deformity, implant displacement, and contour irregularities (33, 34). Over the past decade, there has been a renewed interest in developing and refining PBR (35, 36). This shift is mainly attributed to the limitations of SBR and the enhanced implant support provided by breast reconstruction meshes (37, 38). Moreover, the development and application of mesh have expanded the indications for breast reconstruction, enabling more patients to undergo single-stage procedures that address reconstruction in a single operation, thereby reducing hospitalizations and lowering medical costs. Therefore, owing to their combined advantages, one-stage immediate prepectoral breast reconstruction (PBR) and dual-plane reconstruction are currently preferred by both surgeons and patients. However, due to the absence of large-scale randomized controlled trials, the superiority of either surgical approach remains controversial.

Furthermore, previous meta-analyses have often overlooked the impact of mesh type on clinical outcomes. In clinical practice, the most commonly used meshes include biological meshes, such as acellular dermal matrix (ADM), and synthetic meshes, such as TiLOOP Bra. Although direct head-to-head studies comparing the effects of different meshes on breast reconstruction safety are lacking (22), previous research—including retrospective studies by Katharina et al. and Ohlinger et al., as well as a randomized controlled trial by Gschwantler-Kaulich et al.—has shown that the ADM group demonstrated a significantly poorer safety profile compared with the synthetic mesh group (23, 24, 39). Therefore, the choice of mesh material plays a crucial role in the safety of breast reconstruction and should not be overlooked. Accordingly, ADM was used for all patients in our study. We also compared smoking status, neoadjuvant chemotherapy rate, postoperative adjuvant chemotherapy rate, and postoperative radiotherapy rate between the two groups. These variables are critical determinants of breast reconstruction outcomes. However, they are often overlooked in other related meta-analyses.

Our study was a comprehensive systematic review and meta-analysis of eight published articles. All included reconstructions used ADM and were performed as single-stage immediate implant-based procedures. The study analyzed the overall incidence of complications(including seroma, hematoma, implant loss, capsular contracture, skin or nipple necrosis, wound dehiscence, rippling, and infection)between the prepectoral and subpectoral groups. The pooled analysis indicates that PBR is associated with a lower rates of overall complications These findings are consistent with those of a meta-analysis by Jiameng Liu et al., which included 13 studies comparing prepectoral and subpectoral single-stage breast reconstructions conducted between 2010 and 2020 (OR: 0.54, 95% CI: 0.44–0.67, *p* < 0.001). Although that study did not restrict reconstruction timing (immediate or delayed), SBR modality (fully or partially subpectoral), group characteristics (e.g., smoking status, adjuvant or neoadjuvant chemotherapy), or the type of mesh used, its findings still support, to some extent, the safety of single-stage PBR (40). However, when interpreted in light of the 95% PI, the overall pattern suggests a potential benefit, but the evidence remains insufficient. Because the PI includes 1, the effect across different centers and populations may plausibly range from benefit to no effect, or even a slightly unfavorable effect. These findings should therefore be interpreted cautiously, taking into account the clinical context and the methodological quality of the included studies.

In addition, our study demonstrated that PBR is associated with a lower rate of hematoma. Despite meticulous surgical techniques, the incidence of hematoma after immediate breast reconstruction has been reported in the literature to range from 1% to 7% (41–43). In our analysis, the hematoma incidence was approximately 1.6% in the PBR group and 3.9% in the SBR group. Although postoperative hematoma is a potential complication of any surgical procedure, its occurrence after implant-based breast reconstruction may lead to acute or long-term adverse outcomes. For instance, hematomas increase local pressure, impeding normal wound healing and potentially causing wound dehiscence. The resulting pressure may also displace the implant or alter its shape, compromising the symmetry and natural appearance of the reconstruction. Furthermore, hematomas significantly elevate the risk of capsular contracture, which can lead to breast hardening and deformation, adversely affecting both aesthetics and comfort (44, 45). Simultaneously, hematomas provide an ideal breeding ground for bacteria, readily triggering infections. In severe cases, implant removal may be necessary to control the infection (46).Therefore, minimizing the incidence of hematoma remains an important objective in breast reconstruction. Furthermore, the higher incidence of hematoma observed in the SBR group may be attributed to the deeper placement of the implant between the chest wall and the pectoralis major muscle, where blood supply is abundant. Consequently, muscle movement or contraction may lead to rebleeding (47).In contrast, PBR positions the implant anterior to the pectoralis major muscle, thereby avoiding complications related to muscle movement or contraction. This approach reduces the incidence of hematoma and offers a distinct advantage over SBR in reducing or even eliminating animation deformities (48). Our findings further support these advantages of the PBR technique. Although only two studies were included in the statistical analysis, no animation deformities were reported among 227 PBRs, whereas 352 cases of animation deformity occurred in 523 SBRs. From our results, it seemed that PBR is effective in reducing hematoma incidence and preventing animation deformities. However, it is important to note that, for hematoma incidence under the random-effects model, both the 95% confidence interval (CI) and the 95% prediction interval (PI) crossed the line of no effect, and the PI was extremely wide, indicating that the effect may vary substantially across settings and could plausibly differ in direction. Therefore, the current evidence for this outcome is insufficient, and additional high-quality studies are needed to confirm these findings. Our study also revealed that PBR is associated with a lower rate of skin-nipple necrosis In the study by Zhu and Liu (47), a similar analysis was conducted comparing the incidence of postoperative mastectomy skin-flap necrosis after acellular dermal matrix (ADM)–assisted breast reconstruction performed in the prepectoral versus partially subpectoral plane, and they reported no significant difference. Although both studies suggested that implant placement beneath the pectoralis major may increase the risk of hematoma, our analysis identified a significant difference in the risks of skin necrosis and nipple(-areola complex) necrosis, which may be attributable to differences in patient characteristics, surgical technique, or postoperative care. Importantly, all reconstructions in our study involved immediate, single-stage implant placement, whereas they did not control for the timing of implant insertion. Although other confounders may remain, this key factor may be an important contributor to the observed discrepancies between studies. Nipple or skin ischemia and necrosis are serious complications of implant-based breast reconstruction, with reported incidences ranging from 12.2% to 64.1%. These complications can result in unsatisfactory aesthetic outcomes, delayed wound healing, heightened patient anxiety, implant removal, and postponement of oncologic treatment (49–53). In PBR procedures, nipple and skin flap ischemic necrosis are more likely to cause exposure of the ADM and implant because of the lack of muscle coverage. Therefore, although our meta-analysis demonstrated a significantly lower rate of skin-nipple necrosis in the PBR group compared with the SBR group, understanding and implementing strategies to prevent and minimize this complication remain essential in patients undergoing immediate single-stage prepectoral breast reconstruction. Regarding patient selection, PBR was considered only when the mastectomy skin flaps were of adequate thickness (at least 1 cm) and well perfused (54). Although the optimal thickness of breast reconstruction flaps is difficult to quantify, it is clear that thicker flaps are associated with improved vascularity and enhanced tissue support, whereas thin flaps increase the risk of delayed healing and flap necrosis. Most of our included articles were retrospective studies and the non-randomness of patients between the two cohorts should be noted. Under the random-effects model, the 95% PI analysis indicates that although the overall pooled effect suggests a statistically significant benefit, the PI implies that the true effect in future real-world settings may be inconsistent across populations and centers. Therefore, this conclusion should be generalized with caution. In summary, with proper patient selection, prepectoral or dual-plane placement may be equally safe in terms of early complications.

This study aimed to compare the safety of immediate single-stage prepectoral and partial subpectoral implant-based breast reconstruction using ADM. Although our inclusion criteria were restrictive, all patients in the analyzed studies underwent immediate single-stage breast reconstruction using ADM, which compensates for certain limitations of previous meta-analyses comparing PBR and SBR. Our study also has several limitations. First, the review was not prospectively registered in PROSPERO, which may increase the risk of protocol deviations or selective outcome reporting. To mitigate potential bias, we prespecified the search strategy and inclusion/exclusion criteria before initiating the study. Two reviewers independently performed study screening, data extraction, and quality assessment; any disagreements were resolved through discussion and, when necessary, adjudication by a third reviewer. The review was reported in accordance with the PRISMA guidelines. Secondly, we were able to exclude only a few factors—such as smoking history, neoadjuvant chemotherapy, and postoperative adjuvant chemotherapy—due to the absence of patient-level data. However, other clinically relevant variables (e.g., BMI, diabetes, breast flap thickness, history of radiotherapy) and procedure-related factors (e.g., mastectomy technique, fat processing/injection protocol, implant type, ADM type, extent of coverage (wrap vs anterior sling),drain strategy, and surgeon-related practice patterns) were inconsistently reported or unavailable across studies, precluding subgroup analyses or meta-regression. However, these confounding factors may influence the analysis of surgical safety. In addition, because several included studies reported only that follow-up duration was > 3 months, we were unable to extract more granular follow-up information. Consequently, we could not perform sensitivity analyses stratified by follow-up duration to further assess the impact of follow-up time on complication outcomes. Moreover, insufficient reporting of follow-up characteristics may underestimate delayed complications and the reoperation burden (e.g., delayed implant failure, capsular contracture, and revision/reoperation), thereby limiting our ability to compare long-term safety between approaches (including the finding of similar implant-loss rates).Therefore, it should be cautiously extrapolated to clinical scenarios involving different technical approaches and implant strategies. Moreover, the number of studies included in our analysis was relatively small. Although half of the included studies were prospective cohort designs, no randomized controlled trials were available, making selection bias inevitable. Therefore, well-designed randomized controlled trials are urgently needed to compare the safety and efficacy of immediate single-stage prepectoral and partial subpectoral breast reconstruction using ADM. Furthermore, because wound dehiscence and rippling were each informed by only two studies, the pooled effect estimates are imprecise (with wide confidence intervals), and statistical tests for heterogeneity have limited power. Therefore, these findings should be considered exploratory evidence and require confirmation in additional high-quality studies. In addition, only one included study conducted a comparative analysis of patient satisfaction. Consequently, we were unable to conduct a meta-analysis of patient satisfaction and instead focused solely on safety outcomes. Because some included studies did not report breast-level data, we were required to use the number of patients as the unit of analysis. However, in patients undergoing bilateral reconstruction, this approach may introduce unit-of-analysis bias, because outcomes from the two breasts within the same patient are not fully independent, thereby reducing the precision of the estimates. Bilateral breast outcomes may be correlated, and pooled effect estimates may therefore be affected—particularly for postoperative complications such as hematoma and skin necrosis—potentially leading to underestimation or overestimation of complication risks. To improve analytical precision and the reliability of findings, we recommend that future studies, whenever feasible, prioritize breast-level data and conduct analyses at the breast level to ensure outcome independence and enhance the accuracy of effect estimates. Such an approach would better capture treatment effects for each reconstructed breast and minimize the influence of unit-of-analysis bias. In summary, more well-designed prospective randomized controlled trials are required to provide more robust and comprehensive evidence to guide patients and breast surgeons in selecting the optimal reconstruction approach.

## Conclusion

Our meta-analysis demonstrated that ADM–assisted prepectoral immediate single-stage implant-based breast reconstruction was associated with a lower overall complication rate compared with partial subpectoral reconstruction. Furthermore, PBR exhibited lower incidences of hematoma and skin-nipple necrosis than partial SBR. Therefore, our findings suggest that prepectoral immediate single-stage implant-based breast reconstruction using ADM. was associated with lower safety risks. However, since all studies are observational and long-term efficacy data is extremely limited, these findings may have been influenced by multiple confounding factors; thus, high-quality prospective controlled studies are required to validate and strengthen these conclusions.

## Data Availability

The original contributions presented in the study are included in the article/**Supplementary Material**. Further inquiries can be directed to the corresponding author.
