# Supplementary figures and images for: A systematic review and meta−analysis on the prepectoral and partial subpectoral immediate single−stage Implant-Based Breast Reconstruction Using ADM
Source: Front Oncol. 2026 Feb 27;16:1742423. doi: 10.3389/fonc.2026.1742423 (PMC12982063; doi:10.3389/fonc.2026.1742423)

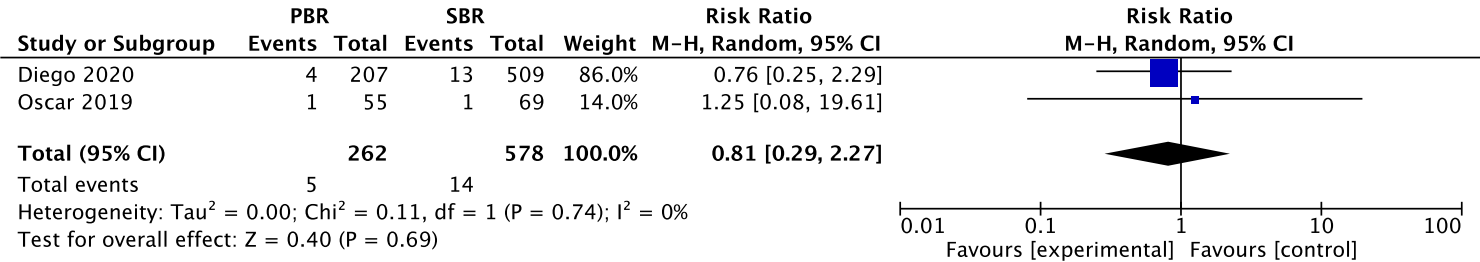

Supplement: Supplementary file 1 [file DataSheet1.zip › Supplementary Material - Results showing both fixed-effect and random-effect models for haematoma and rippling (Forest plot)/Wound dehiscenceΓÇörandom-effects models.pdf]

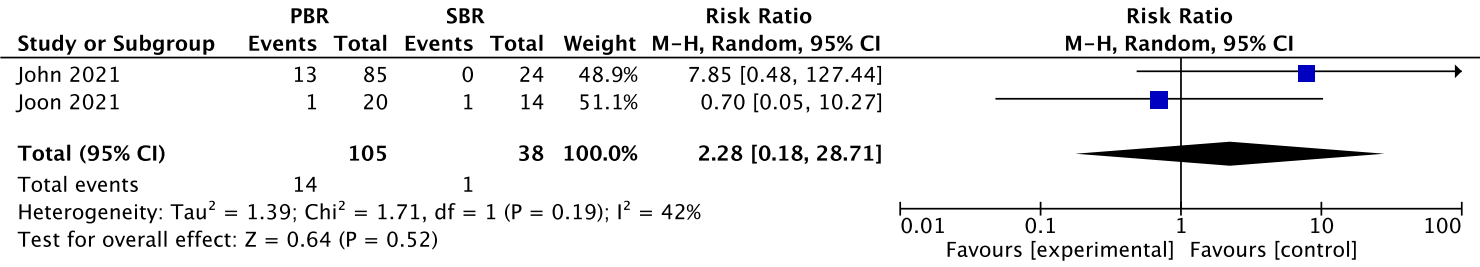

Supplement: Supplementary file 1 [file DataSheet1.zip › Supplementary Material - Results showing both fixed-effect and random-effect models for haematoma and rippling (Forest plot)/RipplingΓÇörandom-effects models.pdf]

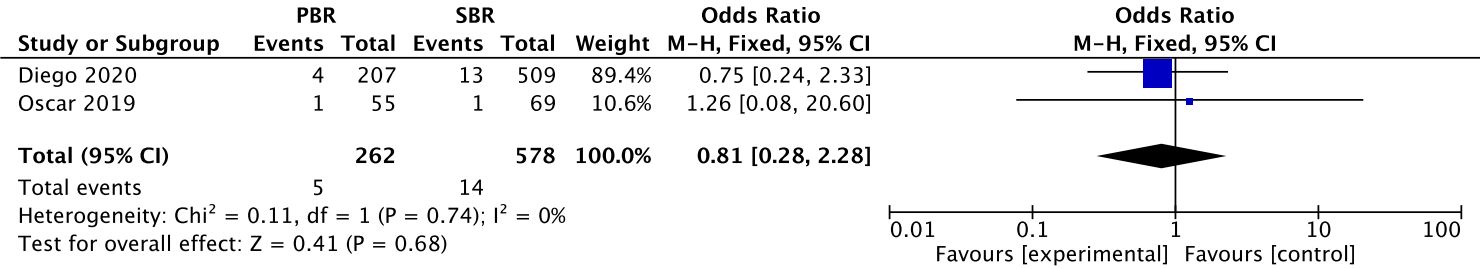

Supplement: Supplementary file 1 [file DataSheet1.zip › Supplementary Material - Results showing both fixed-effect and random-effect models for haematoma and rippling (Forest plot)/Wound dehiscence ΓÇöFixed-effects models.pdf]

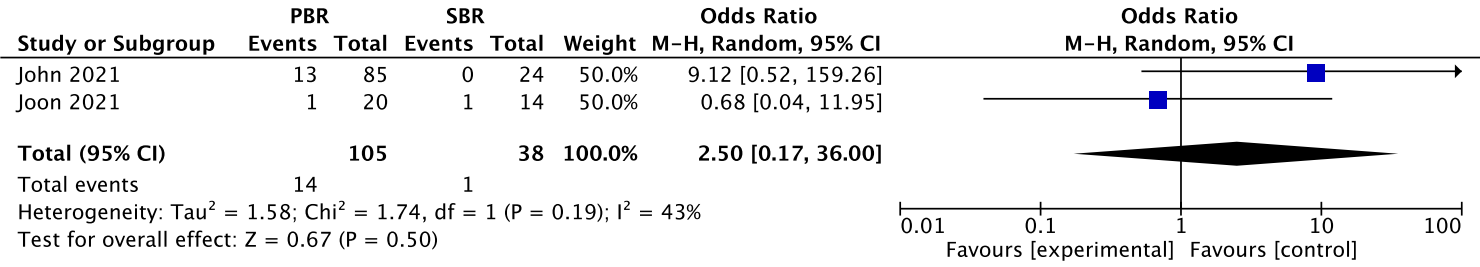

Supplement: Supplementary file 1 [file DataSheet1.zip › Supplementary Material - Results showing both fixed-effect and random-effect models for haematoma and rippling (Forest plot)/RipplingΓÇöFixed-effects models.pdf]
