# Supplementary material for: A systematic review and meta−analysis on the prepectoral and partial subpectoral immediate single−stage Implant-Based Breast Reconstruction Using ADM
Source: Front Oncol. 2026 Feb 27;16:1742423. doi: 10.3389/fonc.2026.1742423 (PMC12982063; doi:10.3389/fonc.2026.1742423)
Supplement: Supplementary file 2 [file Table1.docx]

**Table S2** Search strategy for PubMed/MEDLINE

| Database | PubMed/MEDLINE |
| --- | --- |
| Date run | From relevant studies published to June 21, 2025 |
| MeSH terms and Free-text terms | acellular dermis[MeSH Terms]、acellular dermal matrix、ADM |
|  | mammaplasty[MeSH Terms]、breast reconstruction |
|  | prostheses and implants[MeSH Terms]、implant、prosthesis、prosthetic |
| Free-text terms（No MeSH terms） | prepectoral、suprapectoral、 subcutaneous、premuscular、supramuscular、muscle-sparing、pectoralis-sparing |
|  | subpectoral、submuscular、retropectoral |
| Search query | (((((acellular dermis[MeSH Terms]) OR (acellular dermal matrix)) OR (ADM)) **AND** ((mammaplasty[MeSH Terms]) OR (breast reconstruction))) **AND** ((prepectoral OR suprapectoral OR subcutaneous OR premuscular OR supramuscular OR muscle-sparing OR pectoralis-sparing) **AND** (subpectoral OR submuscular OR retropectoral))) **AND** ((((prostheses and implants[MeSH Terms]) OR (implant)) OR (prosthesis)) OR (prosthetic)) |
| Filters/Limits | No filters or limits were applied during the searches |
